# Keratoconus Diagnostic and Treatment Algorithms Based on Machine-Learning Methods

**DOI:** 10.3390/diagnostics11101933

**Published:** 2021-10-19

**Authors:** Boris Malyugin, Sergej Sakhnov, Svetlana Izmailova, Ernest Boiko, Nadezhda Pozdeyeva, Lyubov Axenova, Kirill Axenov, Aleksej Titov, Anna Terentyeva, Tamriko Zakaraiia, Viktoriya Myasnikova

**Affiliations:** 1 S.N. Fyodorov Eye Microsurgery Complex Federal State Institution, 127-486 Moscow, Russia; malyugin@mntk.ru (B.M.); lana-dok@mail.ru (S.I.); 2 Faculty of Medicine, A. Yevdokimov Moscow State University of Medicine and Dentistry, 127-473 Moscow, Russia; 3 S.N. Fyodorov Eye Microsurgery Complex Federal State Institution, 350-012 Krasnodar, Russia; s_sakhnov@inbox.ru (S.S.); tamriko.zakaraiia@gmail.com (T.Z.); 4 S.N. Fyodorov Eye Microsurgery Complex Federal State Institution, 192-283 Saint-Petersburg, Russia; office@mntk.spb.ru (E.B.); mr.titov@gmail.com (A.T.); 5 S.N. Fyodorov Eye Microsurgery Complex Federal State Institution, 428-027 Cheboksary, Russia; npozdeeva@mail.ru (N.P.); anyaterentieva@yandex.ru (A.T.); 6 Fast Lane, 197136 Saint-Petersburg, Russia; axenov.kir@gmail.com

**Keywords:** keratotopography, keratotomography, keratoconus, data visualisation, classification, machine learning, diagnostics, treatment

## Abstract

The accurate diagnosis of keratoconus, especially in its early stages of development, allows one to utilise timely and proper treatment strategies for slowing the progression of the disease and provide visual rehabilitation. Various keratometry indices and classifications for quantifying the severity of keratoconus have been developed. Today, many of them involve the use of the latest methods of computer processing and data analysis. The main purpose of this work was to develop a machine-learning-based algorithm to precisely determine the stage of keratoconus, allowing optimal management of patients with this disease. A multicentre retrospective study was carried out to obtain a database of patients with keratoconus and to use machine-learning techniques such as principal component analysis and clustering. The created program allows for us to distinguish between a normal state; preclinical keratoconus; and stages 1, 2, 3 and 4 of the disease, with an accuracy in terms of the AUC of 0.95 to 1.00 based on keratotopographer readings, relative to the adapted Amsler–Krumeich algorithm. The predicted stage and additional diagnostic criteria were then used to create a standardised keratoconus management algorithm. We also developed a web-based interface for the algorithm, providing us the opportunity to use the software in a clinical environment.

## 1. Introduction

Keratoconus is a non-inflammatory, progressive, bilateral dystrophic disease characterised by conical corneal protrusion, irregular astigmatism and stromal thinning of the cornea at the apex. The main diagnostic methods for assessing the presence of keratoconus are topography, tomography, pachymetry and biomicroscopy [1]. Corneal topography and Scheimpflug imaging, with a Pentacam system, are used to analyse the corneal surface. This system is based on elevation detection and allows for the assessment of decentration, anterior and posterior surface conditions and corneal pachymetry [2]. The most common problem in the treatment of keratoconus is its diagnosis in its initial stages. Detecting keratoconus at an early stage provides patients with the opportunity to start treatment earlier, thus slowing or even halting the progression of the disease. Patients with formal fruste keratoconus are at high risk of developing iatrogenic ectasia after corneal refractive procedures such as LASIK [3]. Various indices and classifications have been developed to quantify the severity of keratoconus. Among them are TKC parameter and Bellin–Ambrosio ectasia index of the Pentacam system [4]. One of the surgical classifications of keratoconus was proposed in 2014 by Izmailova S.B [5]. It is based on several parameters including corrected visual acuity, maximum keratometry, pachymetry, corneal height maps measured with the Pentacam and biomicroscopy and confocal microscopy data. The most widely used and included in the European clinical guidelines for the treatment of keratoconus [6] is the classification proposed by Amsler in 1946 [7]. It is based on keratometric criteria and includes pachymetric and refractive data. Its modern adapted versions—the Amsler–Krumeich algorithm, 1998 [8], and the George Asimellis algorithm, 2013 [9]—are currently in wide use. A new ABCD classification system was presented by Michael Belin in 2017 [10]. It uses four parameters to assess disease severity: the anterior and posterior radii of curvature, minimum corneal thickness and corrected visual acuity.

With advances in technology and the accumulation of data, more and more studies are showing that it is possible to automate the process of keratoconus diagnosis. Various machine-learning methods are being used for this purpose, which are a subset of artificial intelligence methods. The term “artificial intelligence” was introduced in 1956 by John McCartney and is a general term that “refers to hardware or software that exhibits behaviour which appears intelligent” [11]. Numerous studies using machine-learning techniques to diagnose keratoconus have focused on determining its subclinical form. Indices have been created for Pentacam, Galileo, Sirius and Obscan topographers, with an accuracy of 0.96 for subclinical keratoconus detection [12]. It has also been shown that the use of not only keratotopography data but also parameters of other keratotomography devices can improve diagnostic accuracy [13,14]. In addition to digital device parameters, keratotopographer topographic maps are used to classify eyes with normal topography and eyes with keratoconus [15]. The use of machine-learning techniques can help to both solve the problem of diagnosing keratoconus and predict the course of the disease. For example, Josefi et al. showed that it was possible to predict the probability of keratoplasty using optical coherence tomography data. An unsupervised learning method, such as clustering, was used for this purpose, and the probability of surgery was estimated using the ratio of the number of all the eyes in the cluster to the number of eyes having been operated on [16]. In a study by Velázquez-Blázquez et al., a predictive model for the classification of the initial stages of keratoconus, according to the RETICS scale with an accuracy of 73%, was developed based on a set of demographics, as well as optical, pachometric and morphogeometric variables [17,18].

There are numerous nomograms for the management of patients with keratoconus, which mainly include parameters such as the degree of keratoconus, disease progression and contact lens wearability. For example, in Ismailova’s algorithm, the main factor influencing the choice of treatment tactics is the stage of keratoconus, which is determined using modern diagnostic methods [5]. The algorithms developed by Mohammadpour [19] and Andreanos [20] represent a synthesis of previous studies. They are based on disease progression, corrected visual acuity, corneal thickness and keratometry.

In this paper, we present the results of developing a machine-learning model for the diagnosis of keratoconus, as well as an algorithm that summarises the recommendations for treatment tactics.

## 2. Materials and Methods

### 2.1. Data

Patients’ data from Pentacam devices were automatically obtained as 8 CSV format tables in the S. Fyodorov Eye Microsurgery Complex Head Office (Moscow) and its branches located in Krasnodar, Cheboksary and St. Petersburg during the period from 2015 to 2021. Baseline settings were used in the measurements on the device. After merging the data contained in different tables as well as on different instruments, only the rows with optimal data quality with an “OK” survey status were retained. Data obtained from one eye of the one patient with an “OK” status on the same day were deleted. In the database, which contained all the data from the Pentacam, 47,419 rows remained. Data on keratoconus patients from electronic medical records containing information on the keratoconus stage and visual acuity were collected manually. These data were added and merged with the Pentacam database by patient ID, after which all the data were de-identified. In the final database, 734 rows contained information on the Pentacam measurements, keratoconus stage and visual acuity. The data were processed and analysed using the Python 3 programming language.

This study was performed in accordance with the ethical standards in the Declaration of Helsinki and was approved by the Local Ethical Committee. Informed consent was obtained from the participants. The data protection measures included the de-identification of the data, and the use of local computers to store and process the data within the organisation’s network. In describing this work, we considered the TRIPOD recommendations [21].

### 2.2. Data Labelling and Obtaining the Dataset

As the aim of our study was to determine the stage of keratoconus, the first step was to select a suitable classification. For this purpose, keratoconus stages were added to the Pentacam device readings according to the ABCD classification [10] as well as according to an adapted Amsler–Krumeich (AK) algorithm [9]. The parameters and reference values for these algorithms are shown in Table 1.

The amount of data obtained after applying the classification, as well as from electronic case histories, is presented in Table 2. In addition, after combining keratotopographer data, as well as keratoconus stage data, from electronic medical records, the final database included keratoconus stages that was defined by the ophthalmologists. A sufficient number of rows for training the ML model was obtained only for the AK classification, as it was not possible to use one or seven cases to select the most influential parameters, which included 490 measurements. In this case, the result would be approximate and inaccurate. Therefore, this classification was taken as the basis for further stages of the work. From the result database, we obtained a dataset that contained 400 healthy eyes; 400 eyes with preclinical keratoconus or stages 1, 2 and 3 were selected by random sampling and 52 eyes with stage 4.

### 2.3. Feature Selection

The stages of keratoconus determined by the adapted AK algorithm were a dependent variable for the feature-selection step. In this study, we used StandartScaler for normalisation of independent variables to the range 0–1 and the RFE algorithm with logistic regression as an estimator that provided the importance of features. The estimator updated coefficients that held the fitted parameters. Important features corresponded to high absolute values of the coefficients. We used ‘newton-cg’ as a solver with ‘multinomial’ parameter for the multiclass problem. As a result, out of 490 Pentacam parameters, the 7 most significant parameters were selected using the RFE method. The descriptions of the selected parameters are presented in Table 3.

### 2.4. Machine-Learning Algorithms and Evaluation of Results

**Figure 1:**
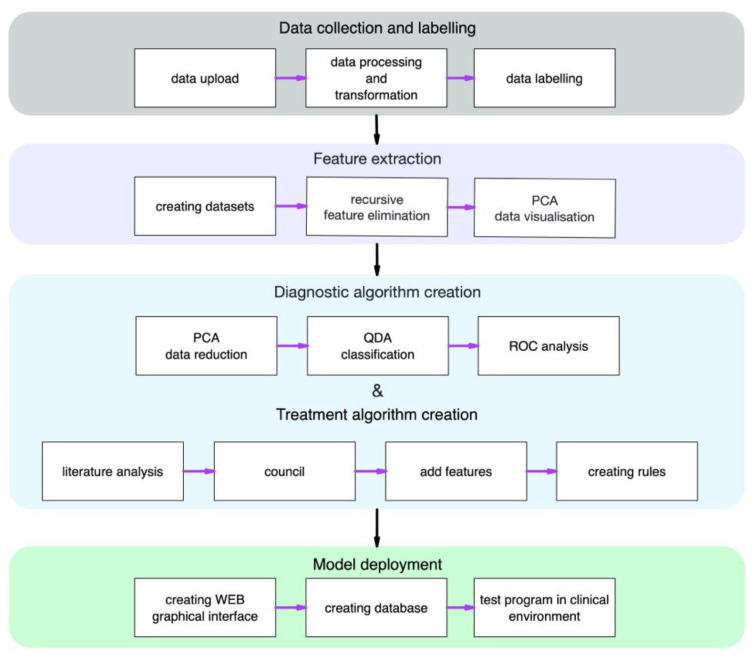
Study design.

For ML model development, we used the same dataset as in Section 2.3, which included only those parameters that had been selected in the feature-selection step. The data were split with a 60:40 ratio: 60% of the data were used for training, and 40%, for testing. First, we used PCA to reduce the dataset dimension from seven parameters to two components and quadratic discriminant analysis (QDA) to classify condition of the patients. Then, we applied the model for test data. To check the quality of the model, we used the AUC metric as well as a visual analysis of the data distribution to compare the resulting model, relative to the adapted AK algorithm. The machine-learning model was developed using Python 3 and the open-source library scikit-learn. Figure 1 is a complete study design for a developed algorithm for the diagnosis and treatment of keratoconus.

## 3. Results

The first step in our work was to determine the stages of keratoconus in the resulting database. For this purpose, we selected two classifications—the ABCD classification developed in 2018 and the adapted AK algorithm. The staging of these classifications resulted in stages 0–4 of keratoconus for AK, as well as stages 1 and 2 for ABCD from the overall database. There were also cases with stages 1 and 2 determined by the ophthalmologists in the database. The number of rows for ABCD and physician-defined stages 3 and 4 was insufficient for further analysis.

Thus, the keratoconus stages determined by the adapted AK algorithm were used to select the most significant parameters; they were the dependent variable for the RFE method. Out of 490 keratotopograph parameters, the 7 parameters most influential for keratoconus stage selection were identified (Table 3).

Table 4 shows the calculated mean values for the parameters selected by RFE. A gradual increase in ISV, IVA, KI, IHD, K Max (Front) and IS-Value and a decrease in R Min from normal to stage 4 keratoconus can be observed.

**Figure 2:**
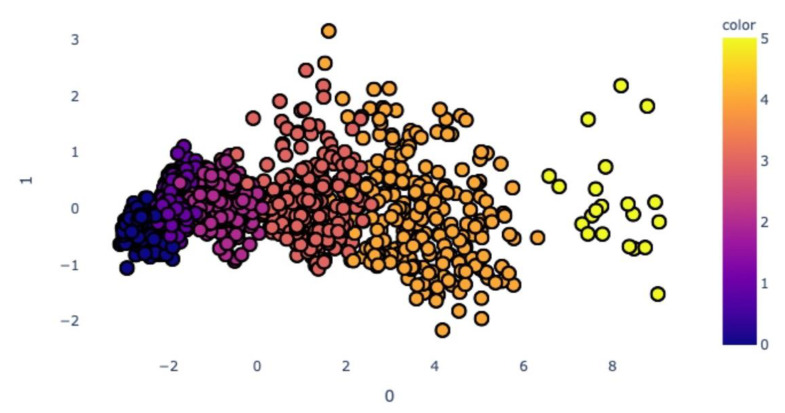
Distribution of the train data on the 2-D plane after the PCA method and coloured according to the stages of keratoconus. Blue—normal; dark purple—preclinical keratoconus; light purple—stage 1; pink—stage 2; orange—stage 3; yellow—stage 4.

The next step of our work was diagnostic algorithm creation. After normalisation of train data, we implemented the PCA method to linear transformation from seven parameters to the two principal components. Figure 2 shows the distribution of the training data, which are coloured according to the stage of keratoconus (normal; preclinical keratoconus; and stages 1, 2, 3 and 4).

**Figure 3:**
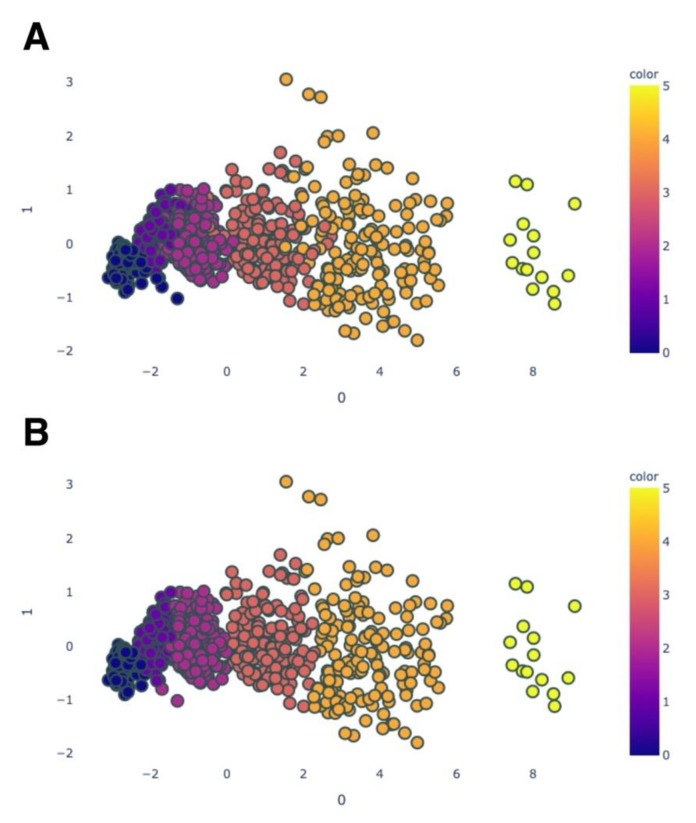
(**A**). Distribution of the test data on the 2-D plane after the PCA method and coloured according to the adopted AK algorithm stages. Blue—normal; dark purple—preclinical keratoconus; light purple—stage 1; pink—stage 2; orange—stage 3; yellow—stage 4. (**B**). Distribution of the test data after the QDA method and coloured according to the predicted stages of keratoconus.

In the next step, the quadratic discriminant analysis was fitted to the training dataset, which consisted of two parameters (principal components) as determined by the PCA method. As a result, we received the classification model for keratoconus stages. This model we applied to predict of the keratoconus stages in test data. Figure 3 visualises the test data after PCA (A) and QDA (B).

**Figure 4:**
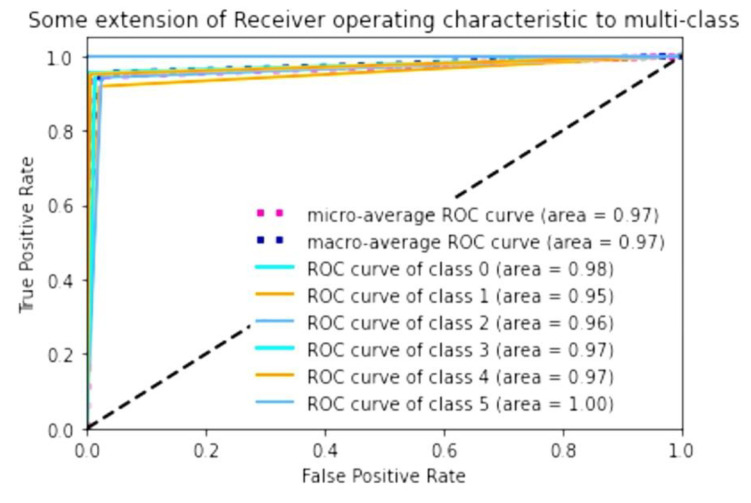
Result of ROC analysis and calculation of AUC (area) for prediction of keratoconus stages from test data relative to the adapted AK algorithm.

To evaluate the accuracy of the algorithm, we calculated the AUC of the prediction of keratoconus stage contained in test data relative to the adapted AK algorithm. It showed high AUC values when performing ROC analysis. The macro-average, which is the result of computing the metric independently for each class and then taking the average and the micro-average, which is the result of aggregating the contributions of all the classes to compute the average metric, is 0.97. If we consider the AUC for a single stage, it was the highest for stage 4 (1.00, class 5). For normal eyes (class 1), it was 0.98; for stages 2 (class 3) and 3 (class 4), it was 0.97; and for stage 1 (class 2), it was 0.96. Preclinical keratoconus (class 1) had the lowest AUC; the result for this group of eyes was 0.95 (Figure 4).

The next step in our work was to create an algorithm for the automated determination of the best keratoconus treatment tactics. For this purpose, additional diagnostic measurements for corneal structure were identified through reviewing the literature and consulting physicians and were used for constructing the algorithm. The parameters and values used in our algorithm to determine the treatment tactics are shown in Table 5. These parameters include the keratoconus stage, determined using our developed machine-learning model, as well as the minimum corneal thickness (measured by a kertotopographer), endothelial cell density (ECD) (biomicroscopy), presence of scarring or opacities (biomicroscopy), ability to correct vision with lenses or glasses (from the medical record), maximum corrected visual acuity (from the medical record) and presence of keratoconus progression (from the medical record).

**Figure 5:**
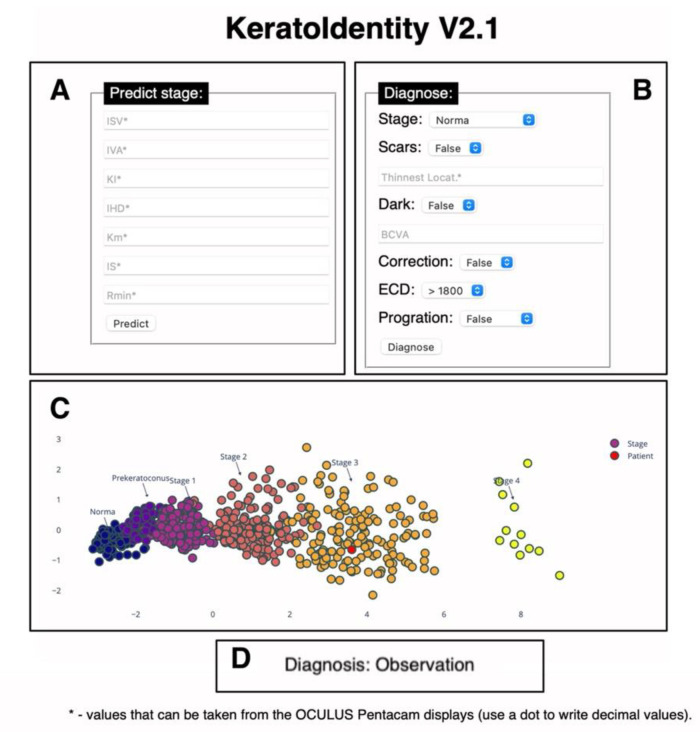
Graphical interface of the software for determining the stage of keratoconus, as well as the indications for surgical intervention: (**A**)—field for manual input of parameters to determine stage of keratoconus; (**B**)—field for manual input of parameters to determine patient management tactics; (**C**)—graphical representation of model including data distribution after PCA and QDA fit (coloured points) as well as new patient data (red points) after PCA and QDA predictions; (**D**)—treatment algorithm result.

A graphical interface was developed for the keratoconus diagnosis model derived from the study as well as the algorithm for determining the indication for surgical intervention. The resulting web application has input fields (Figure 5A,B) for entering information about the parameters measured with the Pentacam, as well as additional diagnostic parameters. The result is displayed as a graphical representation of the model with determined keratoconus stage (Figure 5C) or type of surgical intervention (Figure 5D).

This application has a web form and is located at the following address:

mntk.predictspace.com (the program is not registered as a medical device and is not a substitute for a medical diagnosis).

## 4. Discussion

Studies dedicated to the use of machine-learning techniques have mainly focused on determining the presence of keratoconus, especially its subclinical form [12,14], as treatment of the initial stages is less invasive and helps to avoid further disease progression. The aim of our study was to develop an algorithm for the determination of both the presence of keratoconus and its stages. For this purpose, the first task we had to perform was to choose the most relevant keratotopographer parameters for keratoconus diagnostics. The parameters that have the greatest influence on the stage of keratoconus according to the adapted AK algorithm are the most commonly used in the diagnosis of keratoconus in clinical practice [22].

There are several topographic criteria for diagnosing KC. They can be divided into three main subgroups: curvature-based, elevation-based, and pachymetry-based. The rotating Scheimpflug camera (Pentacam, Oculus GmbH, Wetzlar, Germany) can generate various indices within each of the three index subgroups. The TKC parameter provides the doctor with information about the keratoconus stage. Additionally, information about the stage and status of keratoconus are presented in the extended Belin–Ambrosio ectasia index of the Pentacam. Unfortunately, none of them are 100% sensitive or specific. Some authors believe that elevation maps are better than axial curvature maps for KC screening, while others claim that curvature is still the most sensitive parameter [4].

Earlier studies on keratoconus diagnosis using machine-learning methods have mainly used supervised methods such as support vector machine, random forest and regression analysis. In the present study, we used a supervised machine-learning method, quadratic discriminant analysis (QDA). Accuracy of this method reached an average of 0.97. The best accuracy with the test dataset was shown for stage 4, and the worst for preclinical keratoconus. These data show very similar distribution of keratoconus stages determined by the AK algorithm and stages predicted using the developed model.

The present work has a number of limitations that we plan to solve in our future studies. To build a machine-learning model, we used the keratoconus stages and parameters that were identified during the extraction of the parameters most significant with respect to the adapted AK algorithm. This algorithm is the most widely used, but a newer ABCD classification is now available. In the present study, we obtained datasets for stages 1, 2, 3 and 4 and preclinical keratoconus using the AK algorithm; 1 and 2 using the ABCD classification; and 1 and 2 as defined by ophthalmologists. The ABCD classification, in addition to parameters such as the anterior and posterior radii of curvature and minimum corneal thickness, also uses parameters of corrected visual acuity. Due to the lack of corrected visual acuity data in the electronic database, this parameter was not taken into account when using the classification, which may have resulted in insufficient stages 3 and 4 in the resulting dataset. We plan to add data containing corrected visual acuity information and create a model based on the ABCD classification to compare with this algorithm in the near future.

We also attempted to collect data containing information on the stages of keratoconus diagnosed by clinical physicians. In the obtained dataset, sufficient cases for further analysis were only collected for stages 1 and 2, making it impossible to use this method of determining the keratoconus stage for model development.

Another limitation is that we did not compare the results obtained using the algorithm with the doctor’s decision regarding the choice of treatment tactics. Therefore, we plan to test our algorithm in the clinical environment. We are also planning to collect data about doctors’ decisions and to develop a fully automated model of keratoconus treatment.

Thus, after passing the testing phase and the practical confirmation of high accuracy, the developed software solution could become a complementary diagnostic method and allow the standardisation of the treatment process.

## 5. Conclusions

In summary, we have developed a diagnostic software solution that includes a model for the automated determination of the keratoconus stage, with an accuracy from 0.95 to 1.0, as measured against the adapted AK algorithm. In addition, this software contains a standardised algorithm for determining the indication for surgical intervention, based on data from the literature and recommendations from the expert community. The software has a web interface, which will allow us to adopt it into wider clinical practice and conduct further research in the near future.

## 6. Patents

Certificate of state registration of a computer program No. 2021662273 Russian Federation. Keratoconus Diagnostic and Treatment Program.

## Figures and Tables

**Table 1:** Keratoconus classifications.

**Belin ABCD Keratoconus Grading System** [10]				
	**A** **(ARC)**	**B** **(PRC)**	**C** **(Pachy min)**	D * **(BDVA)**
Stage 0 (normal)	>7.25 mm	>5.9 mm	>490 ϻm	≥20/20
Stage 1	>7.05 mm	>5.7 mm	>450 ϻm	<20/20
Stage 2	>6.35 mm	>5.15 mm	>400 ϻm	<20/40
Stage 3	>6.25 mm	>4.95 mm	>300 ϻm	<20/100
Stage 4	< 6.15 mm	< 4.95 mm	≤300 ϻm	<20/400
**The adapted algorithm, Amsler–Krumeich** [9]				
	**ISV**	**KI**	**R min**	CDVA *
Pre-stage (early signs)	<30	1.04–1.07	7.8–6.7	20/20–20/15
Stage 1	30–55	1.07–1.15	7.5–6.5	20/25–20/15
Stage 2	55–90	1.10–1.25	6.9–5.3	20/60–20/20
Stage 3	90–150	1.15–1.45	6.6–4.8	20/125–20/30
Stage 4	>150	>1.50	<5.00	20/400–20/100

* the parameter was not used because there were not enough data.

**Table 2:** Numbers of rows after applying the AK and ABCD classifications to the resulting database.

	AK	ABCD	Stages from EHR
Normal eyes	7900	23,349	-
Preclinical stage	1369	-	-
Stage 1	1060	41	272
Stage 2	1041	71	235
Stage 3	1225	-	7
Stage 4	52	-	1

**Table 3:** Parameters most relevant in relation to the adapted AK algorithm.

ISV	Index of Surface Variance
IVA	Index of Vertical Asymmetry
KI	Keratoconus Index
IHD	Index of Height Decentration
K Max (Front)	Maximum Curvature Power on Corneal Front Surface
IS-Value	Inferior-Superior Value
R Min (mm)	Minimal Sagittal Curvature

**Table 4:** Mean values, standard deviations, minima and maxima for the parameters included in the model.

	ISV	IVA	KI	IHD	K Max (Front)	IS-Value	R Min (mm)
Normal (Cluster 0)	18.37 ± 9.66 (7.0–82.0)	0.13 ± 0.09 (0.03–0.61)	1.01 ± 0.03 (0.86–1.06)	0.01 ± 0.001 (0–0.05)	42.53 ± 0.96 (39.37–46.8)	0.2 ± 0.73 (-3.22–2.95)	7.97 ± 0.13 (7.81–8.57)
Stage 0 (Cluster 1)	26.72 ± 1.39 (25.0–29.0)	0.19 ± 0.06 (0.06–0.35)	1.05 ± 0.01 (1.04–1.07)	0.02 ± 0.01 (0.0–0.05)	45.87 ± 1.42 (41.83–50.08)	1.00 ± 0.56 (-0.77–2.72)	7.37 ± 0.23 (6.74–7.8)
Stage 1 (Cluster 2)	40.07 ± 6.54 (30.0–54.0)	0.40 ± 0.13 (0.10–0.71)	1.10 ± 0.02 (1.08–1.14)	0.04 ± 0.02 (0.01–0.08)	47.82 ± 1.72 (43.25–52.46)	2.48 ± 0.98 (-0.37–4.94)	7.07 ± 0.25 (6.50–7.50)
Stage 2 (Cluster 3)	71.39 ± 9.32 (55.0–89.0)	0.78 ± 0.18 (0.20–1.21)	1.18 ± 0.03 (1.11–1.24)	0.10 ± 0.03 (0.01–0.17)	53.10 ± 2.95 (48.88–63.13)	5.06 ± 1.50 (0–8.77)	6.38 ± 0.34 (5.35–6.90)
Stage 3 (Cluster 4)	112.81 ± 15.10 (90.0–149.0)	1.24 ± 0.29 (0.23–2.04)	1.32 ± 0.06 (1.16–1.44)	0.17 ± 0.04 (0.02–0.28)	59.12 ± 4.36 (51.28–69.68)	8.82 ± 2.27 (2.01–15.18)	5.72 ± 0.42 (4.84–6.58)
Stage 4 (Cluster 5)	176.36 ± 13.48 (157.0–208.0)	1.74 ± 0.27 (1.27–2.39)	1.60 ± 0.06 (1.51–1.78)	0.31 ± 0.02 (0.26–0.36)	72.66 ± 3.98 (67.99–84.15)	14.99 ± 1.76 (11.03–18.27)	4.66 ± 0.24 (4.01–4.96)

**Table 5:** Parameters and their values used to determine treatment options for keratoconus.

Type of Surgery	-	CXL	ICRS	LPK	PK
KK stage (*)	norm/0/1	1/2	1/2/3	3/4	3/4
Pachymetry min	>400	>400	>400	≤400	≤300 or <1800 or yes/no
ECD	>1800	>1800	>1800	>1800	
Scarring	no	no	no	no	
Blurring	no	no	no	yes/no	yes/no
Possibility of correction	yes	yes/no	yes/no	no	no
BCVA	≥1	≥0.8	<0.8	≤0.2	≤0.2
Disease progression (**)	no	yes	yes/no	yes/no	yes/no

* Keratoconus stage determined by a machine-learning model. ** taken into account when information was available.

## Data Availability

Restrictions apply to the availability of these data. The data were obtained from The S. Fyodorov Eye Microsurgery Complex Federal State Institution and are available from Axenova L. with the permission of the S. Fyodorov Eye Microsurgery Complex Federal State Institution.
